# Type 2 Diabetes and Gastrointestinal Cancers: Risk Associations and Awareness of Screening Challenges

**DOI:** 10.3390/cancers17182989

**Published:** 2025-09-12

**Authors:** Monika Storman, Leszek Czupryniak

**Affiliations:** 1 Department of Internal Medicine, Pulmonary Diseases and Allergy, Medical University of Warsaw, 02-097 Warsaw, Poland; 2 Department of Diabetology and Internal Medicine, Medical University of Warsaw, 02-097 Warsaw, Poland

**Keywords:** type 2 diabetes mellitus, gastrointestinal cancer, screening, colonoscopy, pancreas

## Abstract

Objectives: Type 2 diabetes (T2D) has been increasingly associated with a heightened risk of various gastrointestinal (GI) cancers. This narrative review aims to synthesise current evidence on the link between newly diagnosed T2D and GI malignancies, and to propose a clinical framework for risk-adapted cancer vigilance. Methods: We conducted a narrative review of the literature focusing on the association between T2D and GI cancers, including colorectal, pancreatic, liver, gastric, and biliary malignancies. We examined shared risk factors, underlying biological mechanisms, and emerging insights into pathophysiology. Results: Epidemiological and mechanistic studies suggest that chronic hyperglycaemia, hyperinsulinemia, insulin resistance, inflammation, and gut microbiota alterations contribute to cancer development in patients with T2D. Despite these findings, current screening guidelines do not provide T2D-specific recommendations for GI cancer surveillance. Conclusions: T2D is an emerging risk factor for several GI malignancies. Clinicians should be aware of this association and consider individualised assessment in newly diagnosed patients. The proposed algorithm is intended to stimulate further discussion and guide future research. Prospective studies are needed to evaluate the effectiveness and feasibility of targeted screening strategies in this high-risk population.

## Simple Summary

Patients with type 2 diabetes may face a higher risk of developing certain cancers of the digestive system, such as cancers of the bowel, pancreas, liver, stomach, and bile ducts. This narrative review synthesises current knowledge on how and why this connection may occur. Possible reasons include long-term high blood sugar levels, high insulin levels, chronic inflammation, and alterations in gut bacteria. Despite growing evidence, there are currently no cancer screening guidelines made specifically for patients with type 2 diabetes. We suggest a practical approach to help doctors identify those who might benefit from earlier or more focused checks for digestive system cancers. This could lead to earlier detection and better outcomes. It is intended to prompt discussion among clinicians, researchers, and health policymakers.

## 1. Introduction

Over the past three decades, the global prevalence of type 2 diabetes (T2D) and cancer has increased markedly. Currently, 537 million adults aged 20–79 years are living with diabetes worldwide, with 85–95% of these cases attributable to T2D. This figure is projected to rise to 643 million by 2030 and 783 million by 2045 [1]. In parallel, the incidence of certain cancers has also grown. In 2018, there were an estimated 18.1 million new cancer cases and 9.5 million cancer-related deaths globally. By 2040, these numbers are expected to reach 29.5 million and 16.4 million, respectively.

Gastrointestinal (GI) cancers constitute a substantial proportion of the global cancer burden. Colorectal cancer (CRC) alone accounts for nearly 10% of all new cancer diagnoses worldwide, with approximately 1.9 million new cases each year—equivalent to about 1 in every 4200 individuals—and causes around 935,000 deaths annually. Hepatocellular carcinoma (HCC) represents roughly 900,000 new cases per year, corresponding to about 1 in 9000 people globally, with approximately 830,000 related deaths. Gastric cancer affects about 1 million new patients annually and results in an estimated 770,000 deaths. Pancreatic cancer, one of the most lethal malignancies, is diagnosed in approximately 495,000 people worldwide each year, equivalent to about 1 in 16,000 individuals. Oesophageal cancer affects nearly 600,000 people annually, while gallbladder cancer—though less common—still accounts for approximately 219,000 new cases each year.

Extensive epidemiological studies and meta-analyses have consistently demonstrated an association between T2D and an elevated risk of various gastrointestinal (GI) malignancies [2,3,4]. Specifically, T2D is associated with nearly a twofold higher risk of pancreatic cancer and HCC, a 27% greater risk of colorectal cancer, and a 30% elevated risk of oesophageal cancer. The risk of gallbladder cancer is also increased by more than 50% in this population (Table 1).

The underlying mechanisms linking diabetes and cancer remain incompletely understood but are increasingly clarified by advances in molecular and epidemiological research. Central among these mechanisms are several interrelated biological pathways. Chronic hyperglycemia contributes to oxidative stress through increased production of reactive oxygen species (ROS) and the formation of advanced glycation end-products (AGEs), which interact with their receptor (RAGE) to activate pro-inflammatory and pro-tumorigenic signaling cascades [5,6]. Hyperinsulinemia, a hallmark of insulin resistance in T2D, results in sustained activation of insulin receptors—particularly the mitogenic insulin receptor isoform A—and increases circulating levels of insulin-like growth factor 1 (IGF-1) [7,8]. This heightened IGF-1 activity stimulates downstream pathways such as PI3K/AKT and MAPK/ERK, promoting cellular proliferation, inhibiting apoptosis, and enhancing angiogenesis, which collectively drive oncogenesis. Furthermore, chronic low-grade inflammation characteristic of T2D, mediated by adipose tissue expansion and immune cell infiltration, leads to increased levels of cytokines like IL-6, TNF-α, and C-reactive protein, activating key transcription factors including NF-κB and STAT3 that facilitate neoplastic transformation and tumour progression [9,10,11]. Emerging evidence also highlights the role of gut microbiota dysbiosis and compromised intestinal barrier function in modulating systemic inflammation and metabolic endotoxemia, thereby amplifying cancer risk in patients with T2D [12]. Lastly, regulatory non-coding RNAs, such as microRNAs and long non-coding RNAs, have been implicated in the complex crosstalk between metabolic dysfunction and oncogenic processes [13]. Together, these intertwined pathways provide a multifactorial framework underpinning the increased cancer susceptibility observed in individuals with T2D. The following sections provide an in-depth exploration of these mechanisms and their relevance to gastrointestinal malignancies. These mechanisms are described in detail below.

Given the rising burden of both conditions, a critical question emerges: Should individuals with newly diagnosed T2D undergo additional evaluation for GI malignancies? Although the literature increasingly documents these associations, there remains no clear consensus regarding the utility of targeted cancer screening in patients with newly diagnosed T2D. While colorectal cancer (CRC) screening is broadly recommended and supported by robust evidence in average-risk populations, the value of screening for other GI cancers—such as pancreatic or gastric cancer—among patients with T2D is still a matter of debate [8,14].

This narrative review aims to synthesise current evidence on the association between T2D and GI cancers, assess the strength of the proposed mechanistic and epidemiologic links, and suggest a risk-stratified framework for clinical vigilance.

GI cancers were selected for inclusion based on the following criteria: (a) reported associations with T2D in population-based studies and meta-analyses, (b) plausible biological mechanisms linking T2D with carcinogenesis, and (c) relevance to recent clinical guidelines. Cancers for which evidence is limited, or incidence is exceedingly rare (e.g., anal or small bowel cancers) were excluded.

In May 2024, a narrative literature review was conducted to examine the association between T2D and GI cancers. The search was repeated in April 2025. We searched the PubMed/MEDLINE, Embase, and Cochrane databases using keywords including “type 2 diabetes,” “T2D,” “pancreatic cancer,” “colorectal cancer,” “hepatocellular carcinoma,” “gastric cancer,” “biliary cancer,” and “screening.” Publications in English were considered without strict limitation to the last five years to comprehensively capture relevant evidence. Eligibility criteria included systematic reviews, meta-analyses, and observational cohort studies addressing the relationship between T2D and GI cancers. Case reports and studies without quantitative data were excluded. The objective was to summarise the current state of knowledge rather than to perform a formal meta-analysis.

Although this review is not intended to serve as a formal guideline or systematic review, it seeks to enhance clinical awareness and stimulate further research into appropriate early detection strategies for high-risk patients.

## 2. Pathophysiological Mechanisms Linking Type 2 Diabetes and Gastrointestinal Cancers

### 2.1. Hyperinsulinemia and IGF-1

In T2D, pancreatic β-cell compensation for insulin resistance often results in sustained hyperinsulinemia. Insulin itself has mitogenic properties, particularly via the insulin receptor A isoform (IR-A), which selectively stimulates proliferation relative to metabolic signalling. Insulin also increases liver production of IGF-1 and reduces levels of IGF binding proteins, thereby increasing the bioavailability of IGF-1. The IGF-1 receptor (IGF-1R) activates downstream PI3K-AKT and MAPK/ERK pathways, which drive proliferation, inhibit apoptosis, and support angiogenesis—central features of neoplastic transformation [13,15].

### 2.2. Hyperglycemia and Oxidative Stress

Hyperglycaemia causes oxidative stress by the production of reactive oxygen species (ROS), mitochondrial dysfunction, and the creation of advanced glycation end-products (AGEs). The binding of AGEs to their receptor RAGE stimulates intracellular signalling pathways to promote inflammation and tumour cell proliferation and migration [16]. Hyperglycaemia also changes the expression of tumour suppressors and oncogenes, damages the DNA repair system, and changes the tumour microenvironment [17].

### 2.3. Chronic Inflammation and Pro-Tumour Cytokines

T2D is associated with a persistent low-grade inflammatory state. Adipose tissue expansion in insulin-resistant individuals results in macrophage and immune cell infiltration and the production of proinflammatory cytokines in the form of IL-6, TNF-α, and CRP. These mediators not only exacerbate insulin resistance but also directly induce DNA damage and cellular proliferation and promote a pro-tumorigenic microenvironment [9,10]. Chronic inflammation activates NF-κB signalling and upregulates COX-2 and STAT3, which are often overexpressed in GI cancers [11].

### 2.4. Microbiota Dysbiosis and Intestinal Barrier Dysfunction

Emerging evidence indicates a correlation between T2D and changes in the composition of the gut microbiota (dysbiosis) and in the integrity of the intestine. Dysbiosis can increase bacterial translocation and lipopolysaccharide (LPS) levels in circulation, activating TLR4-mediated inflammation and enhancing tumorigenic signalling in the liver, pancreas, and colon [12]. His gut–liver axis may explain higher incidence rates of hepatic and CRC in patients with T2D.

### 2.5. Molecular Crosstalk: Non-Coding RNAs and Oncogenic Pathways

Recent studies have emphasised the regulatory role of microRNAs (miRNAs) and long non-coding RNAs (lncRNAs) in modulating IGF-1R signalling, insulin sensitivity, and inflammatory responses in both T2D and cancer [13]. These non-coding RNAs can act as oncogenes or tumour suppressors, adding a layer of complexity to the T2D-cancer axis.

## 3. Pancreatic Cancer

The most common type of pancreatic malignancy is pancreatic ductal adenocarcinoma (PDAC). According to the World Health Organization (WHO), pancreatic cancer (PC) is the seventh leading cause of cancer-related mortality worldwide. Early detection remains the most effective strategy for improving long-term survival in affected patients [17].

A variety of factors influence PC risk, including the following:

Age: PC is rare in patients under 45 years, with incidence peaking in the seventh and eighth decades of life.

Gender: A slightly higher incidence is observed in males.

Race/Ethnicity: Increased incidence has been reported among Black populations.

Genetics: Familial aggregation significantly increases risk, especially when ≥3 relatives are affected or if the disease occurs before age 50.

Lifestyle: Tobacco smoking at least doubles the risk of PC. Diets high in animal fat and processed red meat and low in vegetables and folic acid may contribute to carcinogenesis. Obesity is also a well-established risk factor.

Comorbidities: T2D and chronic pancreatitis are independently associated with increased PC risk.

Medications: Some T2D treatments may modulate PC risk. Notably, metformin has been linked to improved survival outcomes in patients with PC [18].

Pancreatic tumours may obstruct the pancreatic duct, cause pancreatic atrophy, and lead to paraneoplastic β-cell dysfunction, collectively impairing insulin secretion and inducing T2D [19,20,21]. Approximately 80% of patients with PC present with impaired glucose tolerance or overt T2D, and long-standing T2D is associated with a twofold increased risk of developing PC compared to the general population [22].

Notably, the relative risk of PC is inversely correlated with the duration of T2D. The incidence of PC peaks shortly after or just before a T2D diagnosis, supporting the hypothesis that in some cases, T2D may be a consequence rather than a cause of pancreatic neoplasia [23]. In elderly patients with newly diagnosed T2D, the risk of sporadic PC is six- to eightfold higher than in the general population [24].

Given these findings, newly diagnosed patients with T2D warrant heightened clinical vigilance. Although routine screening for PC is not currently recommended in this population due to the tumour’s low prevalence, careful risk stratification is advised. Contrast-enhanced multidetector computed tomography (CT) remains the gold standard for early detection [25,26], although repeated radiation exposure poses potential risks. Abdominal ultrasound, while non-invasive, has limited sensitivity and specificity, particularly for early-stage disease.

Consequently, there is an urgent need to develop risk assessment tools for identifying individuals at elevated risk of PC among those with newly diagnosed T2D. Several models have been proposed, including the Health Improvement Network (THIN) score [27] and the Enriching New-Onset Diabetes for Pancreatic Cancer (END-PAC) model [28]. However, none have demonstrated sufficient sensitivity or generalisability to justify widespread screening implementation.

Current research efforts focus on individuals aged >50 years at the time of T2D onset, particularly those with low body mass index, unexplained weight loss, elevated fasting glucose levels, or rapidly worsening glycaemic control. Recent evidence also suggests that patients with newly diagnosed T2D who lack typical risk factors for metabolic syndrome may be at increased risk of PC and might benefit from targeted screening [19,20,21,22,23,24,25,26,27,28,29,30,31].

In high-risk patients, particularly those with a strong family history or genetic predisposition, endoscopic ultrasound may serve as a useful screening modality. Until more definitive evidence becomes available, clinicians should tailor their approach to PC screening based on individual patient risk profiles. Ongoing efforts by groups such as the International Cancer of the Pancreas Screening (CAPS) Consortium provide valuable guidance for risk-based surveillance strategies [32].

## 4. Oesophageal Cancer

According to the World Health Organisation (WHO), oesophageal cancer (EC) is the eighth most common malignancy worldwide, with over 600,000 new cases reported in 2020 [33]. EC comprises two main histological subtypes: oesophageal squamous cell carcinoma (ESCC) and oesophageal adenocarcinoma (EAC), the latter of which is now more commonly observed in many Western countries.

Early symptoms of oesophageal cancer are often nonspecific, leading to delayed diagnosis. Definitive diagnosis is typically established via endoscopic biopsy. Known risk factors for EC include age (predominantly affecting patients over 55 years), male sex, tobacco smoking, alcohol consumption (particularly high-percentage spirits), dietary habits, obesity, prior mediastinal radiotherapy, exposure to caustic chemicals (e.g., acids or alkalis), Plummer–Vinson syndrome, genetically determined palmoplantar keratoderma, achalasia, Barrett’s oesophagus, and a prior history of head and neck cancers, which is associated with increased risk of ESCC [34].

A strong association exists between elevated body mass index (BMI) and the development of EAC. Specifically, patients with a BMI > 30 kg/m^2^ have a significantly increased risk compared to those with a BMI < 22 kg/m^2^ [odds ratio (OR) 16.2; 95% confidence interval (CI): 6.3–41.4]. In contrast, ESCC does not appear to be associated with BMI [35].

Since 1991, studies have suggested a possible link between T2D and EC. A 2016 meta-analysis demonstrated that men with T2D have a significantly increased risk of EC [36]. The pathophysiological mechanisms remain unclear but are thought to involve hyperglycaemia, hyperinsulinemia, insulin resistance, delayed gastric emptying [37,38], gastric hypomotility [39], and obesity-associated metabolic disturbances, which alter hormonal and cytokine profiles [40].

Currently, there is no consensus or recommendation in Europe for routine endoscopic screening for ESCC, as such strategies are not considered cost-effective. Likewise, no general population screening program for EAC has been implemented, largely due to the cost, logistical complexity, need for sedation, and limited evidence of mortality reduction through such interventions [41].

Endoscopic screening for oesophageal cancer may be appropriate only in high-risk individuals, including those with a personal history of head and neck cancer, achalasia, caustic injury, or longstanding gastroesophageal reflux disease (GERD) lasting more than five years, particularly when combined with multiple risk factors such as age > 50 years, white race, male sex, and obesity.

In this context, patients with T2D—especially those with additional risk factors—may benefit from closer clinical surveillance. However, the association between T2D and EC remains contentious, and there is currently insufficient evidence to support the introduction of routine screening in this population. Well-designed prospective studies are urgently needed to investigate the potential link further, ideally accounting for confounding lifestyle factors. Although patients with T2D undoubtedly require comprehensive diagnostic and therapeutic care, no targeted screening protocols for EC have yet been developed for this group.

## 5. Gastric Cancer

According to GLOBOCAN 2018 data, gastric cancer is the fifth most common malignancy and the third leading cause of cancer-related mortality globally. Its incidence is highest in East Asia, Eastern Europe, and parts of South and Central America. Established risk factors include male sex, age over 60 years, *Helicobacter pylori* infection, overweight or obesity, diets high in salt, smoked or pickled foods, alcohol consumption, tobacco smoking, a history of gastric surgery, and genetic predisposition.

Despite a global 5-year survival rate of approximately 24%, mortality from gastric cancer can be reduced by up to 40% through early detection via endoscopic screening. This is supported by screening programs in high-incidence countries such as Japan and South Korea [42].

Epidemiological evidence suggests a potential association between T2D and increased gastric cancer risk [43,44,45,46]. Hyperglycaemia—even before the clinical diagnosis of T2D—has been identified as a possible predictor of gastric cancer [47,48]. Meta-analyses have further indicated that the association between T2D and gastric cancer may be particularly pronounced in women and Asian populations [49].

Despite this evidence, no formal recommendations exist regarding gastric cancer screening in patients with T2D. However, based on current data, it may be prudent to consider screening inpatients over the age of 40 who possess additional risk factors, particularly in regions with intermediate or high gastric cancer incidence.

## 6. Colorectal Cancer

Colorectal cancer is the second leading cause of cancer-related morbidity and mortality in Europe, with an estimated 380,000 new cases and 175,000 related deaths in 2018 [50]. Most CRCs arise from precursor lesions (adenomatous polyps), which can develop along the entire length of the colon and rectum and can be detected by colonoscopy or sigmoidoscopy. Early detection and resection of precancerous polyps by endoscopic examination is the most effective method of preventing CRCs [51] because it is associated with a 69% reduction in disease incidence [52]. Among risk factors are being overweight or obese, not being physically active, high consumption of red meat, smoking, alcohol use, age ≥ 50 years, a personal history of inflammatory bowel disease, a family history of CRC or adenomatous polyps, and having an inherited syndrome [52]. Several studies have suggested that T2D carries an average 30% increased likelihood of developing colorectal malignancy [34,53,54,55,56]. For average-risk populations, ESGE recommends the implementation of organised population-based screening programs based on faecal immunochemical testing (FIT), targeting individuals, irrespective of gender, aged 50–75 years [57]. Several mechanisms have been proposed whereby the increased risk among patients with T2D is associated with the metabolic disorders characteristic of this disease (e.g., hyperglycemia, insulin resistance, hyperinsulinemia or elevated levels of insulin-like growth factor 1—IGF-1) [58]. Additionally, the diabetic microenvironment, such as advanced glycation end-products (AGEs), hyperlipidemia, local inflammation/oxidative stress, changes in the extracellular matrix, microbiome alterations or ischemia due to vasculopathy, may activate secondary injury mediators that may promote colorectal carcinogenesis as well as T2D complications [59]. Another proposed mechanism involves the existence of T2D susceptibility genes and colorectal neoplasia, suggesting potentially common pathogenetic pathways such as *TCF7L2*, *KCNQ1*, *HMGA2*, *RHPN2*, and *GREM1* [60]. T2D is associated with an increased risk of CRC, particularly in adults < 50 years, and risk-adapted CRC screening based on personal T2D history, with and without a family history of CRC, may be beneficial [61]. Accordingly, a colonoscopy may be considered in patients with T2D 5–10 years earlier than in the general population.

## 7. Hepatocellular Carcinoma

Hepatocellular carcinoma (HCC) is the most common primary malignancy of the liver and one of the leading causes of cancer-related mortality worldwide. The risk of HCC is significantly elevated in T2D, particularly in those with a long-standing disease duration, defined in several cohort studies as exceeding 10 years. This risk appears to be independent of—but frequently amplified by—coexisting factors such as chronic viral hepatitis (HBV or HCV), obesity, and alcohol consumption [62,63].

Recent studies have identified metabolic dysfunction-associated steatotic liver disease (MASLD), formerly referred to as non-alcoholic fatty liver disease (NAFLD), as a critical component in the pathophysiologic continuum linking T2D to HCC. MASLD is now recognised as a common hepatic complication of T2D, affecting up to 70% of patients with T2D [64]. The progression from MASLD to metabolic steatohepatitis (MASH), advanced fibrosis, and eventually HCC is increasingly well-documented and represents a non-cirrhotic pathway to hepatocarcinogenesis [65,66].

In patients with viral hepatitis, T2D is acknowledged as a modifier of disease progression. A cohort study from Taiwan demonstrated that newly diagnosed T2D significantly increased the risk of HCC in patients with HBV-positive (relative risk: 1.63; 95% CI: 1.11–2.38) [67]. Similar synergistic effects have been observed in patients with alcohol-related liver disease and chronic hepatitis C virus (HCV) infection, suggesting that T2D enhances hepatocarcinogenic signalling across various forms of liver injury [68].

Given these findings, it is advisable to perform baseline testing for hepatitis B surface antigen (HBsAg) and anti-HCV antibodies in patients newly diagnosed with T2D, especially in those with obesity or a history of alcohol consumption. Moreover, abdominal ultrasonography may be considered in selected patients with hepatic risk factors, although routine screening for HCC remains limited to individuals with cirrhosis or advanced fibrosis.

## 8. Gallbladder Cancer and Cholangiocarcinoma

Gallbladder cancer (GBC) is relatively rare; however, it accounts for approximately 80–95% of all cholangiocarcinomas and is the fifth most common cancer of the digestive system [69]. Established risk factors for GBC include chronic gallstones, gallbladder polyps (particularly those exceeding 1 cm in diameter), biliary tract cysts, chronic calcifying cholecystitis, and increasing age—most diagnosed in patients over 50 years. The biological relationship between T2D and GBC likely involves hyperinsulinaemia, hyperglycaemia, and elevated concentrations of insulin-like growth factor 1 (IGF-1). A meta-analysis of 20 observational studies published in 2015 confirmed an increased risk of GBC in patients with T2D compared to non-diabetic controls [70].

Cholangiocarcinoma (CC) can be classified into intrahepatic and extrahepatic subtypes. The overall prognosis remains dismal, with reported 5-year survival rates typically below 10%. Well-recognised determinants of CC include primary sclerosing cholangitis (PSC), long-standing intraductal gallstones, liver fluke infections, choledochal cysts, Caroli’s disease (congenital intrahepatic cystic lesions), adenoma of the biliary tract, biliary papillomatosis, exposure to Thorotrast, inflammatory bowel disease (IBD), and persistent typhoid carriage [71]. Putative contributors comprise chronic viral hepatitis B and C, obesity, type 2 diabetes, non-alcoholic fatty liver disease, excessive alcohol consumption, tobacco use, genetic polymorphisms, chronic inflammation, and alterations in biliary transporter function. The association between T2D and CC remains unclear [72].

Some studies have identified T2D as an independent risk factor for cholelithiasis [73], which is itself a major risk factor for CC. Other investigations, particularly those conducted in Asian populations, suggest that T2D may be a pathogenic contributor to the development of CC [74].

It appears that all patients over the age of 50 with newly diagnosed T2D and additional risk factors for gallstones should undergo initial abdominal ultrasonography.

## 9. The Potential Harms of Increased Screening

Increased screening for GI cancer in patients with newly diagnosed T2D can offer the benefit of early diagnosis but is also linked with several potential harms, including overdiagnosis, patient anxiety, cost implications, and the risks associated with specific procedures.

Overdiagnosis occurs when screening tests identify cancers that would not have manifested as symptoms or death during the lifetime of the patient. This can result in unnecessary treatment, exposure to side effects and emotional distress from the procedure of diagnosis/treatment. For example, colonoscopy and FOBT can detect slow-growing or benign polyps, thus exposing patients to excessive treatment for cancer that they will likely never experience.

Screening and cancer diagnosis are universally anxiety-provoking and emotionally distressing, and as many as 56% of patients undergoing examinations for GI cancer develop mild or moderate anxiety. Anxiety can peak with the initial outpatient visit and recur with surgery or invasive procedures [75].

Routine screening of all patients with newly diagnosed type 2 diabetes increases health costs due to more frequent tests, follow-up procedures, overtreatment, and longer-term monitoring. It may overwhelm health systems if screenings find low-risk or non-progressive cancers [76].

It is also important to acknowledge the risks inherent in these procedures. Some endoscopic procedures (e.g., colonoscopy or sigmoidoscopy) have risks including bleeding, perforation, infection, and reactions to anaesthetics. These risks are potentially worsened in patients with T2D because they tend to have comorbid illnesses like obesity, cardiovascular disease, or impaired wound healing, all of which can increase procedural complications.

## 10. Conclusions

The association between T2D and GI cancers is increasingly recognised and supported by plausible biological mechanisms, including insulin resistance, chronic inflammation, and hyperinsulinemia, as well as consistent epidemiological signals, particularly in the case of colorectal and pancreatic cancers. However, the clinical implications of this association remain to be clearly defined.

At present, CRC screening remains the sole approach with demonstrated efficacy, and it is broadly endorsed for the general population, encompassing patients with T2D. For other GI malignancies, such as pancreatic, hepatic, or gastric cancers, the evidence supporting population-level screening remains insufficient. Furthermore, the relatively low absolute risk and potential harms of procedures like endoscopy, including rare but serious complications such as bleeding or cardiopulmonary events, underscore the need for caution.

## 11. Future Directions

The ADA Standards of Medical Care in Diabetes advise that patients undergo age- and sex-appropriate cancer screenings. Beyond these general recommendations, however, no formal guidelines exist for tailored oncological surveillance specifically in patients with newly diagnosed T2D. While certain subgroups—such as patients over 50 with low BMI, sudden weight loss, or rapidly worsening glycemia—may warrant more vigilant monitoring, any proposed screening strategies should be individualized based on risk stratification rather than universal application.

**Figure 1:**
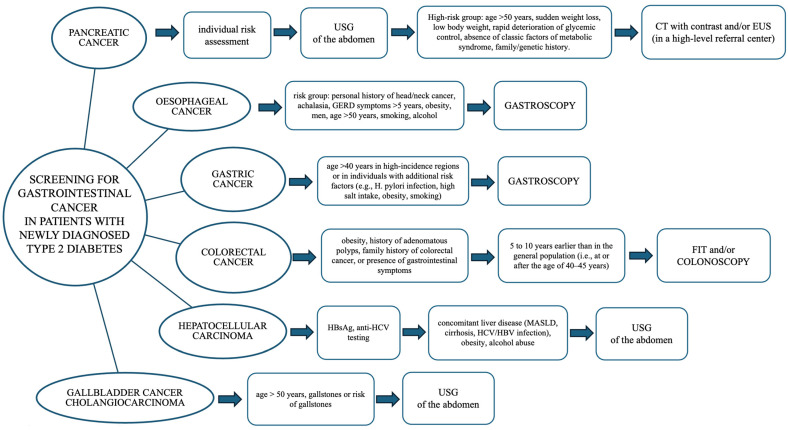
Suggested screening approach in new-onset type 2 diabetes.

Our proposed algorithm (Figure 1) represents a structured and cautious approach to stratified vigilance in newly diagnosed patients with T2D. However, we emphasise that this tool is exploratory and should not be interpreted as a formal recommendation. Further prospective, population-based studies are essential to validate such approaches. These studies should evaluate diagnostic yield, safety, cost-effectiveness, and, ultimately, clinical outcomes. Until then, raising clinician awareness of potential cancer risks in patients with T2D, while avoiding overdiagnosis and overtreatment, remains a balanced and responsible path forward.

## Figures and Tables

**Table 1:** Random effects estimate with 95% confidence and prediction intervals from 27 meta-analyses of type 2 diabetes mellitus and cancer incidence (modified from [4]).

Type of Cancer	No. of Cases	Random Effects (95% CI)
Gastric cancer	15,970	1.09 (0.98–1.22)
Colorectal cancer	61,690	1.27 (1.21–1.34)
Hepatocellular carcinoma	33,765	2.31 (1.97–2.84)
Esophageal cancer	3001	1.30 (1.12–1.50)
Gallbladder cancer	1821	1.52 (1.26–1.84)
Extrahepatic cholangiocarcinoma	2431	1.63 (1.29–2.05)
Intrahepatic cholangiocarcinoma	3152	1.97 (1.57–2.46)
Pancreatic cancer	52,445	1.95 (1.66–2.28)

## Data Availability

Available from the authors upon request via e-mail.

## Abbreviations

The following abbreviations are used in this manuscript:

T2D	type 2 diabetes
GI	gastrointestinal
CRC	colorectal cancer
ROS	reactive oxygen species
AGE	advanced glycation end-products
LPS	lipopolysaccharide
PDAC	pancreatic ductal adenocarcinoma
WHO	World Health Organization
PC	pancreatic cancer
CT	computed tomography
EC	oesophageal cancer
ESCC	oesophageal squamous cell carcinoma
EAC	oesophageal adenocarcinoma
OR	odds ratio
CI	confidence interval
GERD	gastroesophageal reflux disease
HCC	Hepatocellular carcinoma
MASLD	metabolic dysfunction-associated steatotic liver disease
NAFLD	non-alcoholic fatty liver disease
GBC	Gallbladder Cancer
CC	Cholangiocarcinoma

## References

[1] International Diabetes Federation (2021). Diabetes Atlas.

[2] Gallagher E.J., LeRoith D. (2013). Epidemiology and molecular mechanisms tying obesity, diabetes, and the metabolic syndrome with cancer. Diabetes Care.

[3] Srivastava S.P., Goodwin J.E. (2020). Cancer biology and prevention in diabetes. Cells.

[4] Tsilidis K.K., Kasimis J.C., Lopez D.S., Ntzani E.E., Ioannidis J.P. (2015). Type 2 diabetes and cancer: Umbrella review of meta-analyses of observational studies. BMJ.

[5] Noto H. (2018). Unfolding link between diabetes and cancer. J. Diabetes Investig..

[6] Ryu T.Y., Park J., Scherer P.E. (2014). Hyperglycemia as a risk factor for cancer progression. Diabetes Metab. J..

[7] Murphy N., Jenab M., Gunter M.J. (2018). Adiposity and gastrointestinal cancers: Epidemiology, mechanisms and future directions. Nat. Rev. Gastroenterol. Hepatol..

[8] Li Z., Chen H., Fritz C.D.L., Wu C., Li M., Ma X., Huang J., Ge Z., Cai S., Zheng S. (2022). Type 2 diabetes and risk of early-onset colorectal cancer. Gastro Hep Adv..

[9] Tilg H., Moschen A.R. (2014). Mechanisms behind the link between obesity and gastrointestinal cancers. Best Pract. Res. Clin. Gastroenterol..

[10] Amin M.N., Hussain M.S., Sarwar M.S., Rahman M.M., Islam M.S., Jahan M., Islam M.N., Islam S.M.A., Mouly T.A., Ahmed K. (2019). How the association between obesity and inflammation may lead to insulin resistance and cancer. Diabetes Metab. Syndr..

[11] Kasprzak A. (2021). Insulin-like growth factor 1 (IGF-1) signaling in glucose metabolism in colorectal cancer. Int. J. Mol. Sci..

[12] Moodi M., Tavakoli T., Tahergorabi Z. (2021). Crossroad between obesity and gastrointestinal cancers: A review of molecular mechanisms. Int. J. Prev. Med..

[13] Chen B., Li J., Chi D., Wang Y., Yu Q., Wang D., Zhang X., Song X., Wang W., Wang Z. (2019). Non-coding RNAs in IGF-1R signaling regulation: The underlying pathophysiological link between diabetes and cancer. Cells.

[14] de Jong R.G.P.J., Peeters P.J.H.L., Burden A.M., Barendregt J.J., McDonald J.A. (2018). Gastrointestinal cancer incidence in type 2 diabetes mellitus: Results from a large population-based cohort study in the UK. Cancer Epidemiol..

[15] Chiefari E., Mirabelli M., La Vignera S., Colao A., Foti D. (2021). Insulin resistance and cancer: In search for a causal link. Int. J. Mol. Sci..

[16] Vella V., Lappano R., Bonavita E., Barbieri A., Maggiolini M., Cirillo F., Leone M.C., Malaguarnera R., Belfiore A., Mauro L. (2023). Insulin/IGF axis and the Receptor for Advanced Glycation End Products: Role in meta-inflammation and potential in cancer therapy. Endocr. Rev..

[17] Szukiewicz D. (2023). Molecular mechanisms for the vicious cycle between insulin resistance and the inflammatory response in obesity. Int. J. Mol. Sci..

[18] Xin W., Fang L., Fang Q., Chen C., Chen H., Ma X., Li M., Wu C., Zhang X., Gao Q. (2018). Effects of metformin on survival outcomes of pancreatic cancer patients with diabetes: A meta-analysis. Mol. Clin. Oncol..

[19] Pelaez-Luna M., Takahashi N., Fletcher J.G., Chari S.T. (2007). Resectability of presymptomatic pancreatic cancer and its relationship to onset of diabetes: A retrospective review of CT scans and fasting glucose values prior to diagnosis. Am. J. Gastroenterol..

[20] Basso D., Plebani M., Fogar P., Mazza A., Zambon C.F., Navaglia F., Scillitani G., Franchin G., Fincati K., Greco E. (1994). Beta-cell function in pancreatic adenocarcinoma. Pancreas.

[21] Li D., Tang H., Hassan M.M., Holly E.A., Bracci P.M., Silverman D.T., Risch H.A., Goodman M.T., Gallinger S., Cotterchio M. (2011). Diabetes and risk of pancreatic cancer: A pooled analysis of three large case-control studies. Cancer Causes Control..

[22] Permert J., Ihse I., Jorfeldt L., Rydberg L., Arnelo U., Dahlen G., Nystrom A., Andersson S.W., Larsson J. (1993). Pancreatic cancer is associated with impaired glucose metabolism. Eur. J. Surg..

[23] Ben Q., Xu M., Ning X., Liu J., Hong S., Huang W., Han X., Wang L., Du Y., Yuan Y. (2011). Diabetes mellitus and risk of pancreatic cancer: A meta-analysis of cohort studies. Eur. J. Cancer.

[24] Chari S.T., Leibson C.L., Rabe K.G., Ransom J., de Andrade M., Petersen G.M. (2005). Probability of pancreatic cancer following diabetes: A population-based study. Gastroenterology.

[25] Kauhanen S.P., Komar G., Seppänen M.P., Dean K.I., Minn H., Kajander S., Rinta-Kiikka I., Alanen K., Oikonen V., Sipilä H. (2009). A prospective diagnostic accuracy study of 18F-FDG PET/CT, multidetector row CT, and MRI in primary diagnosis and staging of pancreatic cancer. Ann. Surg..

[26] Sheridan M.B., Ward J., Guthrie J.A., Robinson P.J., Davies M.H., Hudson M., Moore N.R., Ryder W.D., Carroll N., Marples M. (1999). Dynamic contrast-enhanced MR imaging and dual-phase helical CT in the preoperative assessment of suspected pancreatic cancer: A comparative study with ROC analysis. AJR Am. J. Roentgenol..

[27] Boursi B., Finkelman B., Giantonio B.J., Haynes K., Mamtani R., Yang Y.X. (2022). A clinical prediction model to assess risk for pancreatic cancer among patients with pre-diabetes. Eur. J. Gastroenterol. Hepatol..

[28] Sharma A., Kandlakunta H., Nagpal S.J.S., Bertin P., Gupta R., Chari S.T., Petersen G.M., Butturini G., Piciucchi M., Baron T.H. (2018). Model to determine risk of pancreatic cancer in patients with new-onset diabetes. Gastroenterology.

[29] Ogawa Y., Tanaka M., Inoue K., Yamaguchi Y., Moriya T., Yamamoto T., Yamaguchi K., Matoba K., Satake K., Sekimoto M. (2002). A prospective pancreatographic study of the prevalence of pancreatic carcinoma in patients with diabetes mellitus. Cancer.

[30] Illés D., Terzin V., Holzinger G., Tajti M., Svébis M., Horváth V., Oláh I., Tóth I., Márta K., Váncsa S. (2016). New-onset type 2 diabetes mellitus—A high-risk group suitable for the screening of pancreatic cancer?. Pancreatology.

[31] Molina-Montes E., Gómez-Rubio P., Malats N. (2018). Type 2 diabetes mellitus and pancreatic cancer risk: An independent etiological relation?. Pancreatology.

[32] Canto M.I., Harinck F., Hruban R.H., Offerhaus G.J., Poley J.W., Kamel I., Nio Y., Schulick R.S., Bassi C., Kluijt I. (2014). International Cancer of the Pancreas Screening (CAPS) Consortium summit on the management of patients with increased risk for familial pancreatic cancer. Gut.

[33] International Agency for Research on Cancer Global Cancer Observatory: Cancer Today.

[34] Polish National Cancer Registry. https://onkologia.org.pl.

[35] Lagergren J., Bergström R., Nyrén O. (1999). Association between body mass and adenocarcinoma of the esophagus and gastric cardia. Ann. Intern. Med..

[36] Xu B., Zhou X., Li X., Liu C., Yang C. (2017). Diabetes mellitus carries a risk of esophageal cancer: A meta-analysis. Medicine.

[37] Mearin F., Malagelada J.R. (1995). Gastroparesis and dyspepsia in patients with diabetes mellitus. Eur. J. Gastroenterol. Hepatol..

[38] Kamiya T., Adachi H., Hirako M., Shikano M., Matsuhisa E., Wada T., Ogasawara N., Nojiri S., Kataoka H., Sasaki M. (2009). Impaired gastric motility and its relationship to reflux symptoms in patients with nonerosive gastroesophageal reflux disease. J. Gastroenterol..

[39] Demeester S.R. (2009). Epidemiology and biology of esophageal cancer. Gastrointest. Cancer Res..

[40] Huang W., Ren H., Ben Q., Cai Q., Zhu W., Li Z. (2012). Risk of esophageal cancer in diabetes mellitus: A meta-analysis of observational studies. Cancer Causes Control.

[41] National Cancer Institute Esophageal Cancer Screening. American Cancer Society. https://cancer.gov.

[42] Zhang X., Li M., Chen S., Hu J., Guo Q., Liu R., Zheng H., Jin Z., Yuan Y., Xi Y. (2018). Endoscopic Screening in Asian Countries Is Associated With Reduced Gastric Cancer Mortality: A Meta-analysis and Systematic Review. Gastroenterology.

[43] Ge Z., Ben Q., Qian J., Wang Y., Li Y. (2011). Diabetes mellitus and risk of gastric cancer: A systematic review and meta-analysis of observational studies. Eur. J. Gastroenterol. Hepatol..

[44] Marimuthu S.P., Vijayaragavan P., Moysich K.B., Jayaprakash V. (2011). Diabetes mellitus and gastric carcinoma: Is there an association?. J. Carcinog..

[45] Tian T., Zhang L.Q., Ma X.H., Zhou J.N., Shen J. (2012). Diabetes mellitus and incidence and mortality of gastric cancer: A meta-analysis. Exp. Clin. Endocrinol. Diabetes.

[46] Yoon J.M., Son K.Y., Eom C.S., Durrance D., Park S.M. (2013). Pre-existing diabetes mellitus increases the risk of gastric cancer: A meta-analysis. World J. Gastroenterol..

[47] Ikeda F., Doi Y., Yonemoto K., Ninomiya T., Kubo M., Shikata K., Hata J., Tanizaki Y., Matsumoto T., Iida M. (2009). Hyperglycemia increases risk of gastric cancer posed by Helicobacter pylori infection: A population-based cohort study. Gastroenterology.

[48] Yamagata H., Kiyohara Y., Nakamura S., Kubo M., Tanizaki Y., Matsumoto T., Tanaka K., Kato I., Shirota T., Iida M. (2005). Impact of fasting plasma glucose levels on gastric cancer incidence in a general Japanese population: The Hisayama study. Diabetes Care.

[49] Tseng C.H., Tseng F.H. (2014). Diabetes and gastric cancer: The potential links. World J. Gastroenterol..

[50] International Agency for Research on Cancer. IARC. https://gco.iarc.fr.

[51] Winawer S.J., Zauber A.G., Ho M.N., O’Brien M.J., Gottlieb L.S., Sternberg S.S., Waye J.D., Schapiro M., Bond J.H., Panish J.F. (1993). Prevention of colorectal cancer by colonoscopic polypectomy: The National Polyp Study Workgroup. N. Engl. J. Med..

[52] Brenner H., Stock C., Hoffmeister M. (2014). Effect of screening sigmoidoscopy and screening colonoscopy on colorectal cancer incidence and mortality: Systematic review and meta-analysis of randomized controlled trials and observational studies. BMJ.

[53] Deng L., Gui Z., Zhao L., Wang J., Shen L. (2012). Diabetes mellitus and the incidence of colorectal cancer: An updated systematic review and meta-analysis. Dig. Dis. Sci..

[54] Suh S., Kang M., Kim M.Y., Chung H.S., Kim S.K., Hur K.Y., Kim J.H., Lee M.S., Lee M.K., Kim K.W. (2011). Korean type 2 diabetes patients have multiple adenomatous polyps compared to non-diabetic controls. J. Korean Med. Sci..

[55] Wu L., Yu C., Jiang H., Miao X., Jiang J., Zhou Y., Shi Y., Xiao H., Liang M., Xie L. (2013). Diabetes mellitus and the occurrence of colorectal cancer: An updated meta-analysis of cohort studies. Diabetes Technol. Ther..

[56] Yuhara H., Steinmaus C., Cohen S.E., Corley D.A., Tei Y., Buffler P.A. (2011). Is diabetes mellitus an independent risk factor for colon cancer and rectal cancer?. Am. J. Gastroenterol..

[57] Hassan C., East J., Radaelli F., Spada C., Benamouzig R., Bisschops R., Bretthauer M., Dekker E., Dinis-Ribeiro M., Ferlitsch M. (2019). Bowel preparation for colonoscopy: European Society of Gastrointestinal Endoscopy (ESGE) Guideline—Update 2019. Endoscopy.

[58] Chang C.K., Ulrich C.M. (2003). Hyperinsulinaemia and hyperglycaemia: Possible risk factors of colorectal cancer among diabetic patients. Diabetologia.

[59] Del Puerto-Nevado L., Santiago-Hernandez A., Solanes-Casado S., Gonzalez N., Ricote M., Corton M., Prieto I., Mas S., Sanz A.B., Aguilera O. (2019). Diabetes-mediated promotion of colon mucosa carcinogenesis is associated with mitochondrial dysfunction. Mol. Oncol..

[60] li Khan U., Fallah M., Sundquist K., Hemminki K., Sundquist J. (2020). Risk of colorectal cancer in patients with diabetes mellitus: A Swedish nationwide cohort study. PLoS Med..

[61] American Diabetes Association (2021). Standards of medical care in diabetes—2022. Diabetes Care.

[62] Zhou J., Sun H., Wang Z., Wang Z., Wu G., Chen Q., Wu L., Tang Y., Jiang J., Xie D. (2023). Synergistic interaction of diabetes and hepatitis B on liver cancer risk: Meta-analysis. Cancer Epidemiol..

[63] Younossi Z.M., Golabi P., Paik J.M., Henry A., Van Dongen C., Henry L. (2023). The global epidemiology of nonalcoholic fatty liver disease (NAFLD) and nonalcoholic steatohepatitis (NASH): A systematic review. Hepatology.

[64] Chalasani N., Younossi Z., Lavine J.E., Charlton M., Cusi K., Rinella M., Harrison S.A., Brunt E.M., Sanyal A.J., American Association for the Study of Liver Diseases (2021). The diagnosis and management of MASLD: Practice guidance from the AASLD. Hepatology.

[65] Petroni M.L., Brodosi L., Marchignoli F., Marchesini G. (2024). MASLD-related HCC: Non-cirrhotic pathways and predictive markers. J. Hepatol..

[66] Loomba R., Lim J.K., Patton H. (2021). MASLD progression to HCC: A global perspective. Gastroenterology.

[67] Chen Y., Wu C., Huang Y., Zhang L., Zhu Y., Zhang Z., Shen P., Zhang J., Wu D., Wang L. (2022). Type 2 diabetes and hepatocellular carcinoma risk among hepatitis B patients: A population-based cohort study. Liver Int..

[68] Wong R.J., Cheung R., Ahmed A. (2020). Diabetes and the risk of hepatocellular carcinoma in chronic liver diseases. Hepatology.

[69] Hundal R., Shaffer E.A. (2014). Gallbladder cancer: Epidemiology and outcome. Clin. Epidemiol..

[70] Gu J., Yan S., Wang B., Chen X., He J., Kang N., Wu Q., He X., Jin Y., Wen Y. (2016). Type 2 diabetes mellitus and risk of gallbladder cancer: A systematic review and meta-analysis of observational studies. Diabetes Metab. Res. Rev..

[71] Tyson G.L., El-Serag H.B. (2011). Risk factors for cholangiocarcinoma. Hepatology.

[72] Noel M.S., Hezel A.F. (2013). New and emerging treatment options for biliary tract cancer. OncoTargets Ther..

[73] Biddinger S.B., Haas J.T., Yu B.B., Xue B., Claussnitzer M., Clee S.M., Möbius-Winkler M., Allayee H., Burris T.P., Bachmann O.P. (2008). Hepatic insulin resistance directly promotes the formation of cholesterol gallstones. Nat. Med..

[74] El-Serag H.B., Engels E.A., Landgren O., Chiao E.Y., Gillison M.L., Goodman M.T., Pfeiffer R.M., Weir H.K., Murphy G., Schymura M.J. (2009). Risk of hepatobiliary and pancreatic cancers after hepatitis C virus infection: A population-based study of U.S. veterans. Hepatology.

[75] Harms J., Kunzmann B., Bredereke J., Harms L., Jungbluth T., Zimmermann T. (2023). Anxiety in patients with gastrointestinal cancer undergoing primary surgery. J. Cancer Res. Clin. Oncol..

[76] Suh S., Kim K.W. (2019). Diabetes and Cancer: Cancer Should Be Screened in Routine Diabetes Assessment. Diabetes Metab. J..

